# Patient experiences of telephone outreach to enhance uptake of NHS Health Checks in more deprived communities and minority ethnic groups: A qualitative interview study

**DOI:** 10.1111/hex.12856

**Published:** 2018-12-25

**Authors:** Emer Brangan, Tracey J. Stone, Amanda Chappell, Vivienne Harrison, Jeremy Horwood

**Affiliations:** ^1^ The National Institute for Health Research Collaboration for Leadership in Applied Health Research and Care West (NIHR CLAHRC West) at University Hospitals Bristol NHS Foundation Trust Bristol UK; ^2^ The Centre for Academic Primary Care, Population Health Sciences Bristol Medical School University of Bristol Bristol UK; ^3^ Public Health Bristol City Council Bristol UK

**Keywords:** cardiovascular diseases, ethnicity, inequalities, NHS Health Checks, outreach, qualitative research, socioeconomic factors, telephone invitation

## Abstract

**Background:**

The NHS Health Checks preventative programme aims to reduce cardiovascular morbidity across England. To improve equity in uptake, telephone outreach was developed in Bristol, involving community workers telephoning patients amongst communities potentially at higher risk of cardiovascular disease and/or less likely to take up a written invitation, to engage them with NHS Health Checks. Where possible, caller cultural background/main language is matched with that of the patient called. The call includes an invitation to book an NHS Health Check appointment, lifestyle questions from the Health Check, and signposting to lifestyle services.

**Objective:**

To explore the experiences of patients who received an outreach call.

**Design/Setting/Participants:**

Thematic analysis of semi‐structured interviews with 24 patients (15 female), from seven primary care practices, who had received an outreach call.

**Results:**

The call increased participants’ understanding of NHS Health Checks and overcame anticipated difficulties with making an appointment. Half reported that they would not have booked if only invited by letter. The cultural identity/language skills of the caller were important in facilitating the interaction for some who might otherwise encounter language or cultural barriers. The inclusion of lifestyle questions and signposting prompted a minority to make lifestyle changes.

**Conclusions:**

Participants valued easily generalizable aspects of the intervention—a telephone invitation with ability to book during the call—and reported that it prompted acceptance of an NHS Health Check. A caller who shared their main language/cultural background was important for a minority of participants, and improved targeting of this would be beneficial.

## 1 BACKGROUND

The NHS (UK National Health Service) Health Check programme, introduced in 2009, became a statutory public health service in England in 2013. Local authorities are responsible for offering an NHS Health Check every 5 years to individuals aged 40‐74 who are not on a relevant disease register. The programme aims to prevent heart disease, stroke, type two diabetes and kidney disease, using a combination of risk assessment, communication of risk and risk management.^1^ The programme is one of the largest public health prevention programmes in the world, with over 6 million people in England having received a Health Check since 2013.^1^ Currently the main providers of NHS Health Checks are primary care practices, although they have also been offered in a range of community settings.^2^, ^3^, ^4^ Regardless of provider, primary care follow up any risks identified.^1^

Critiques of the programme have included the risk of widening health inequalities,^5^ with concerns amongst primary care clinicians that it attracts the “worried well,” and that those who could benefit most were least likely to attend.^6^, ^7^ An evaluation of implementation of NHS Health Checks in North West England found support amongst GPs for targeting people expected to be at high risk.^8^ This brings with it a requirement to define “high‐risk” individuals or groups, identify them locally and find methods of increasing the number who attend health checks.

Socio‐economic deprivation is associated with increased morbidity and mortality from cardiovascular diseases.^9^, ^10^ Cardiovascular risk is also known to vary for different ethnic groups, with, for example, South Asians bearing a disproportionate burden of heart disease.^11^

Several studies have looked at NHS Health Check coverage (the percentage of people who are eligible for an NHS Health Check who have received one) or uptake (the percentage of those invited for an NHS Health Check who receive one) in relation to deprivation or ethnicity. While those from the least deprived areas are most likely to take up an invitation to an NHS Health Check, coverage was consistently found to be higher in more deprived areas, which may reflect existing targeting.^3^ Coverage amongst different ethnic groups was also found to be comparable to, or higher than, that in “White British” groups.^3^ However, evidence on uptake in different ethnic groups was mixed, with conflicting findings.^3^ Analyses were limited by high levels of missing ethnicity data in primary care practice records, with uptake significantly lower for those with this data missing.^12^, ^13^ Qualitative research with staff delivering health checks found perceptions that people from black and minority ethnic groups were less likely to attend, with language and cultural issues seen as major barriers.^14^

Various methods aimed at increasing uptake of invitations to NHS Health Checks have been investigated. A review of evidence found modified invitation letters^15^, ^16^, ^17^, ^18^ and use of text messages^15^ to be promising methods for the general eligible population.^3^ However, it is of particular interest to understand how to increase uptake amongst groups who may be at higher risk of cardiovascular disease.

The limited evidence on the effectiveness of invitations to NHS Health Checks by telephone suggests they may increase uptake compared with invitations by letter^19^ or may increase the number of health checks completed for patients from deprived areas or minority ethnic groups.^3^, ^20^ Qualitative research with primary care staff delivering health checks found that some practices were using telephone calls to build on existing relationships with patients or to target those who had not responded to a written invitation.^14^ Qualitative research has also explored the involvement of community ambassadors/engagement workers to increase uptake in specific communities. Reported benefits included their ability to communicate using language people understood and connected with^2^, ^3^, ^21^ and, where the ambassador/worker involved was known and trusted, their endorsement of the health check influenced people to attend.^2^

Telephone outreach has been developed in Bristol which involves specially trained community workers or interpreting service staff telephoning patients amongst communities where people may be at higher risk of cardiovascular disease, and/or less likely to take up a written invitation, to engage them with the NHS Health Checks programme. The intervention was intended, where possible, to match outreach caller cultural background and main language with that of the patient called. The outreach call includes an invitation to book an appointment for a health check, and if this is accepted, the lifestyle questions (eg, on physical activity, smoking and alcohol consumption) from the health check are completed on the telephone, with the aim of saving time during the face‐to‐face health check appointment. Where appropriate, outreach callers may also signpost people to local lifestyle services, based on responses to the lifestyle questions. Telephone outreach has been piloted in ten primary care practices in Bristol, with targeting of eligible registered patients who are identified as residing in areas of high deprivation or as potentially requiring cultural or language support.

Bristol is a culturally and ethnically diverse city, with 16% of the population from black and minority ethnic groups, and 15% of residents having been born outside the UK. Nine per cent of Bristol residents do not speak English as their main language.^22^ The gap in healthy life expectancy between the most deprived and least deprived 10% within Bristol places the local authority area in the worst quintile in England, at 16.3 years for men and 16.7 years for women. Cardiovascular disease is the largest cause of years of life lived in less than ideal health or lost due to premature mortality in Bristol.^22^ The objective of the telephone outreach intervention was to engage people from communities with potentially higher health need with the NHS Health Checks programme, to help reduce inequalities in health.

The objective of this study was to explore in depth the experiences and perspectives of patients who received a telephone outreach call to invite them to take part in an NHS Health Check. Findings from a qualitative evaluation carried out with staff delivering the outreach calls are reported elsewhere (T. J. Stone, E. Brangan, A. Chappell, V. Harrison, J. Horwood, unpublished data).

## 2 METHODS

Patients from seven primary care practices who had received an outreach telephone call were asked at the end of the call whether they were willing to be contacted by a researcher to discuss taking part in this study. Those who agreed to being contacted were sampled purposefully to maximize diversity regarding primary care practice, outreach caller, NHS Health Check invitation acceptance status, age, gender and ethnicity and invited to take part in a semi‐structured interview.^23^ Sample size was driven by the concept of “information power,^24^” with continuous assessment as data collection progressed of the adequacy of the information within our sample with regard to meeting our study objective.

All interviews were carried out by EB and took place by telephone or face‐to‐face according to participants’ preferences. Participants whose main language was not English had the option of an interpreter being present to facilitate the interview. Interviews lasted between 8 and 54 minutes. A topic guide was used to focus the interviews, informed by a review of relevant literature and suggestions from our multiprofessional study team, and modified as data analysis progressed (please see Appendix S1).

With informed consent, interviews were audio recorded, transcribed verbatim, anonymized and imported into NVivo 10 (QSR International). Transcripts were analysed thematically^25^ using a data‐driven inductive approach to identify patterns and themes of particular salience for participants and across the data set.

Analysis began alongside data collection, with ideas from early analysis informing later data collection. Analysis of individual transcripts commenced with open coding and an initial coding framework was developed, which was added to and refined as new data were gathered. A subset of 12% of the transcripts were double coded by EB and JH to inform the coding framework and ensure robust analysis. Codes were built into broader categories through comparison across transcripts and higher‐level recurring themes were developed (please see Appendix S2). Members of the study team met regularly to discuss emerging themes, and the public health professionals responsible for commissioning the local Health Checks programme and management of the telephone outreach project (AC and VH) were closely involved throughout research design, data collection and analysis.

To assist with the development of the study, three patients from local areas of high deprivation and with experience of receiving a telephone outreach call for NHS Health Checks were recruited via existing primary care patient feedback groups. They met with EB and TS, as well as an independent facilitator and a translator, and reviewed the draft study documentation, recruitment procedures and topic guide. The feedback they provided was used to refine the design of the study patient information sheet, as well as the procedures for telephoning patients who had agreed to being contacted about the research.

Informed consent was obtained and documented for all participants to participate in the study and for anonymized quotes to be used in publications reporting the study findings. Written consent was obtained for face‐to‐face interviews. Participants who chose to be interviewed by telephone provided verbal consent. This was documented at the beginning of the interview by audio‐recording the participant verbally confirming their agreement to each of the points contained in the written consent form. All participants were provided with study information in writing a minimum of 1 week before giving consent. The study, including the consent and anonymization procedures used, was approved by the South West‐Frenchay NHS research ethics committee (Reference 15/SW/0231).

## 3 RESULTS

Information about the study was sent by post to 50 patients (33 women) who received a telephone outreach call. This written study information was followed up with telephone contact by a researcher a week later. Thirteen patients (nine female) declined to participate in the study, either by returning a postal opt‐out slip or verbally when follow‐up contact was made by phone. For 13 further patients (nine female), it was either not possible to establish follow‐up contact by telephone, despite repeated attempts, or an interview could not be arranged/completed within the study timeframe. The remaining 24 patients (15 women, nine men, 40‐66 years of age) took part in an interview between March and July 2016. All were recorded as having accepted the invitation to attend an NHS Health Check—while we wished to include the views of people who had declined the invitation to the NHS Health Check, none of those who agreed to being contacted by a researcher were in this category. Seventeen of those interviewed resided in the most deprived quintile by postcode, with a further three in the second most deprived quintile.^26^ Sixteen participants categorized themselves as White British, with other self‐reported ethnicities being Black Caribbean, Black mixed, Bangladeshi, Somali, Jamaican and Polish. Five participants did not have English as their main language, and three chose to be interviewed with the assistance of an interpreter—two Bengali speakers and one Somali speaker.

Results are organized into three overall themes developed from the analysis. “Receiving an NHS Health Check invitation by telephone” presents data regarding the acceptability to participants of the outreach call, and views on benefits or disadvantages compared with other methods of invitation. “Who calls, and how they communicate” focuses on what participants viewed as important regarding the identity, and communication skills and strategies, of the outreach caller. The final theme explores participants’ views and experiences of carrying out part of the NHS Health Check during the call. Pseudonyms are used in reporting the verbatim quotes below.

Approximately a third of participants were aware of history of conditions relevant to NHS Health Checks in close relatives at relatively young ages—for example, stroke or heart attack in their 50s or 60s. A further third either reported that relevant conditions had been in relatives in their late seventies or older or the ages of onset were not clear. The remaining third were not aware of any relevant family history.

The outreach call was the first time 15 of the participants had heard about NHS Health Checks. Seven further participants had heard something about NHS Health Checks but did not have clear/accurate information, and two participants had a clear understanding prior to the call.

Seven different outreach callers made the outreach calls to those interviewed. Two had Somali as their main language, one had Bengali, and the remaining four had English.

### 3.1 Receiving an NHS Health Check invitation by telephone

Participants were pleased to be proactively contacted by telephone and offered a health check.

*I was actually very pleased because, you know, I reached a milestone in my life and health system is inviting me for some checks. I thought, you know, it's just a good care*. *(Janek, 40)*

The majority of participants said that they did not think anything needed to change about how the outreach calls were made. However, five participants mentioned that they would be less likely to answer a call if it came from an unknown number. Three female participants reported some minor initial concern regarding an unexpected call from their health centre.

*That was a bit confusing. Cos you know, your doctor's number comes up and you're kind of thinking ‘why are they phoning me?’* *(Jess, 41)*

Lucy recalled upfront reassurance that there was no cause for concern:

*It was pretty straightforward. Straightaway she said there's nothing to worry about….Put you at ease*. *(Lucy, 60)*

Many participants said that they had accepted the invitation for the NHS Health Check because it seemed intuitively to be a good thing to do and that they did not need much more information or persuasion to accept the invitation:

*Well, it makes sense. And it, sort of, I can't see any reason why not, why wouldn't you?* *(Joseph, 60)*

Despite this, when asked what they would have done if they had been invited by letter rather than via the outreach call, half of the 18 participants who expressed a clear view on the matter said that it was unlikely that they would have made an appointment. Several of these said that they would not even have read the letter fully:

*No I probably wouldn't have read it [laughs]. I probably would have just read the first few lines, probably would have just binned it*. *(David, 40)*

Others would have intended to make an appointment but would not have got around to it:

*If it's not compulsory you can sort of please yourself and you think ‘oh I'll make it next week’ – it's like I had a text from the dentist last week to make an appointment but I haven't rung ‘em up to make one. Do you know what I mean? Whereas I spoke to her [outreach caller] on the phone and she made the appointment there and then, then obviously I went……whereas if they sort of send you a letter ‘do you wanna come for a health check?’ you think ‘no I can't be bothered’…You've gotta put yourself out sort of thing innit?* *(Sharon, 61)*

The ease and immediacy of being able to book an appointment during the outreach call was a key benefit for most participants:

*She actually made the appointment there and then…. and the first one she came up with was, in fact, it was quicker than I thought it was going to be*. *(George, 66)*

Some participants had found it useful to speak to someone about the NHS Health Check rather than receiving a letter, mentioning the ability to ask questions.

Only three participants expressed a preference for other forms of communication. Jennifer, 61, was glad to have been invited but would have preferred a letter, putting this down to being “*a bit old fashioned*.” Rosie was also pleased to have been invited and said that the outreach call had alerted her to the opportunity. She did however request that the information be sent to her in writing:

*Because it was not easy, I just want to sit and read and know what it's all about……Sometime I don't like to talk on the phone, those things, you know, it's either face‐to‐face or you know you get it in writing*. *(Rosie, 60)*

Suleymaan (56) liked to have information about appointments in writing as his wife used this to remind him to attend. He valued the idea of having someone from his own community who could take the time to explain the health check, but, like Rosie, he preferred face‐to‐face conversations to the telephone.

A number of participants mentioned that, after booking their appointments during the outreach call, they had received confirmation by text or letter; thus, the outreach call did not preclude providing written information to meet some of these preferences.

A third of participants mentioned having had a health concern on their mind at the time of the outreach call but stated that they would not have initiated contact with the health service to address this. They welcomed the call to invite them for an NHS Health Check, which they saw as either addressing those concerns directly and/or as an opportunity to discuss their concerns with a health professional.

*My husband has blood pressure problems and I used to think, ‘Well, perhaps I've got blood pressure problems and I should go and have a health check.’ But because I'm not bad, I didn't feel I ought to go…… because if I'm not bad, I didn't feel I could ring up and say I want a health check so I didn't do anything about it*. *(Jennifer, 61)*

### 3.2 Who telephones, and how they communicate

Participants noted the outreach caller's connection to their primary care practice, but beyond this, caller identity was not presented as an important factor in most interviews. However, particular communication skills, or aspects of the identity of the caller, were presented by participants as having facilitated, or occasionally hindered, the interaction to a range of degrees.

Friendliness and a lack of duress in how the invitation was extended were mentioned by the greatest number of participants, but these were presented as helpful rather than essential aspects:

*She was very warm, she was very chatty… it wasn't like you must come for this, she was really chatty so yeh that obviously helps*. *(Liliya, 40)*

Caller identity and communication skills were of greatest importance to three male participants whose main language was not English. Maderu (55) shared Bengali as a main language with the outreach worker who called him and valued being able to deal more directly with the health service through her, rather than having his daughter act as an intermediary. Dananjay said that, as well as sharing a main language, it had been better for him to be called by somebody he knew:

*It was better because I knew that person so I didn't ask many questions. If it was somebody unknown I would have asked many more questions*. *(Interpreter/Dananjay, 50, main language Bengali)*

Suleymaan's (56, main language Somali) views on what was important for him in an outreach call overlapped with and extended those expressed by Dananjay:

*Interpreter/Suleymaan:* 
*I’d probably prefer someone I have seen before*.

*Interviewer:* *Okay. And can you think of any reason why that might be?*

*Interpreter/Suleymaan:* *I suppose, it's I'm going to worry more about someone I don't know at all. If I see the person or if I know the person, then it makes it easier for me to be able to communicate with them, because I don't have to worry so much of, ‘Who is this person? What are they going to be asking you?’ You know, it's just about being anxious about the person that would take a lot of my thinking*.

There were also data which suggested that there could be a downside to a known caller. There were indications that Suleymaan might not be well disposed towards a particular outreach worker who he mentioned by name. Joseph (60) suggested that it might be easier to talk about potentially sensitive topics to someone who he did not know and that it would be easier over the telephone:

*In fact, it's easier isn't it, if it's not face‐to‐face?…..you don't have to… there's no embarrassment or anything, because you don't even know who you're talking to do you?* *(Joseph, 60)*

However, most callers were not already known to the participant, and interviews illustrated diverse experiences of the communication which had occurred during the outreach call. Sonia related how the outreach caller she had spoken to had told her about her own experience of having a health check and that she had found this helpful:

*She said her and her husband had had theirs [NHS Health Checks] done and it was quite reassuring because they found out that her husband had high blood pressure so…somebody that sort of I suppose had already been through it and knew… what they were talking about really herself*. *(Sonia, 41)*

Martin received his outreach call while queuing at his primary care practice and informed his caller of this. He felt uncomfortable having the conversation in that context:

*It was a shame that I was in a slight rush, you know, to get out of people's way and try to listen to this phone call and do everything. I'm not the greatest of persons for that… so if they would have either phoned back or say ‘I'll phone you back later’ or whatever, that would have been great*. *(Martin, 60)*

Four participants commented that the caller had explained things in a way which was easy to understand; however, six participants either said that little information had been provided about the NHS Health Check or that they had not understood/taken in the information. Four of these did not seem perturbed by this, but two men felt somewhat uncertain before their appointments about what would be involved.

Three participants raised the topic of outreach callers with accents/main languages other than English and came across as finding this potentially problematic:

*Interviewer* *The questions you were asked about physical activity and smoking and so on, how was it doing those on the phone?*

*Jess* *It was quite difficult actually. Both people I spoke to’s first language wasn't English, which made it a bit difficult at times*.

However, for Janek (main language Polish), what he described as his caller's “*strong accent*” was mitigated by Janek's acknowledgement that he himself had an accent and that he had recognized the caller's voice from previous encounters at his primary care practice:

*Well I never judge people's accent because I got really strong accent and my English is not perfect by any means. But no, no I don't think that was a barrier…Especially since I knew the guy. I had the face attached to the voice so it was different*. *(Janek, 40)*

### 3.3 Completing part of the health check during the outreach call

The telephone outreach intervention included completing part of the NHS Health Check during the telephone call by asking patients who accepted the invitation to an NHS Health Check about their weight, relevant family health history, smoking status, alcohol consumption and physical activity. Most participants said that it was acceptable to be asked these types of questions on the telephone. While several participants mentioned that they had been asked the questions again at their NHS Health Check appointment, most did not consider this a problem, and two said that this had been a brief check of the information recorded during the outreach call.

Two participants reported a negative experience related to this part of the telephone call. Jess described her experience of being asked the questions as “*quite difficult,*” partly because the caller's main language was not English, but also because she thought the caller was not being receptive to her responses:

*They asked me questions and I think they just expect you to say ‘…I've got one of my parents…’ or something and obviously I had quite an extensive list of people for them, and they kind of then were cutting me off! … if they just wanted me to say ‘yes there's a history of that’ … that would've helped, rather than them asking me ‘who’ and then saying ‘oh ok’ ‘ok, ok’, you know, If you didn't want all of them you shouldn't've asked!* *(Jess, 41)*

Jennifer (61) thought that the way the questions were asked implied that she was too old to be active:

*Jennifer* *Well, funny enough it was on my birthday. I wasn't feeling terribly well because I had a headache and I was a bit shirty with the woman that rang because I was actually walking the dog at the time and she asked me if I was active still. ……..She actually asked me how old I was, how heavy I was, whether I did exercise, whether I smoked, whether I drank and whether I would class myself as still able to do activities in the house and gardening*.

*Interviewer* *Okay. How did you feel about having those kinds of questions over the phone?*

*Jennifer* *Well, to be honest I always think it's a bit rude because I don't consider myself old. So the fact that I'm asked if I'm still active and can still garden, makes me a bit cross sometimes*.

*Interviewer* *Okay, because you feel like maybe that implied that…*

*Jennifer* *I'm ancient*.

As a follow‐up to information disclosed in response to such questions, the telephone outreach intervention includes outreach workers, where appropriate, signposting patients to local lifestyle services. One participant recalled having discussed this during the outreach call:

*She told me about some local sort of fitness clubs, and she actually sent me some details in the post as well. So…that was sort of quite helpful really*. *(Margaret, 62)*

Four participants, including Margaret, mentioned that the lifestyle questions had prompted them to think or act differently:

*I mean I think since I retired, I generally have more exercise now than I did when I was at work. I tend to ‐ I enjoy walking and things like that but I think she did get me thinking ‘oh perhaps I ought to do more than I am doing’*. *(Margaret, 62)*

Dananjay and Rosie reported having made changes in their lifestyle in response to the outreach telephone call. In Dananjay's case, the advice had been reinforced at his NHS Health Check appointment, where the outreach worker who had called him was also present.

*Yes, I took her advice and I have just started walking daily and also she advised me about my diet. I'm also doing it*. *(Interpreter/Dananjay, 50)*

Rosie reported having doubled her physical activity—from one long walk a week to two—before she had gone to the actual health check:

*From I get their call yes I just oh I am gonna look about myself but I was looking about it but now it pushed me more*. *(Rosie, 60)*

## 4 DISCUSSION

The telephone outreach intervention was positively received by the patients interviewed, with the majority reporting that they did not need much information or persuasion to accept the invitation to an NHS Health Check. Participants reported that the ease and immediacy of being able to book an appointment during the outreach call was a key factor in taking up the invitation. This finding is consistent with existing literature. For example, participants attending community‐based health checks^21^ reported a preference for telephone or in‐person invitations, as they were seen as more “immediate and direct,” as well as allowing them to ask questions—a benefit also mentioned by some of our participants.

The outreach callers were given motivational interviewing training as part of their preparation for delivering the telephone outreach intervention, to improve their ability to help participants overcome the intention‐behaviour gap. Previous interventions to increase engagement with NHS Health Checks have used insights from behavioural science to overcome the intention‐behaviour gap and have been shown to be effective.^18^ Participants in our study highlighted that the telephone outreach call had simultaneously increased their knowledge/understanding of the NHS Health Checks programme and overcome anticipated difficulties with making an appointment—both getting around to trying, and the process once they did try.

These aspects of the intervention—providing a telephone call with information about, an invitation to, and an opportunity to book, an NHS Health Check—would be easily generalizable, as they could be carried out by primary care administration staff. This approach could also potentially be used for other services/interventions to increase uptake.

Language and cultural issues have been reported previously by staff delivering NHS Health Checks as major barriers to engaging with minority ethnic groups.^14^ The Bristol telephone outreach intervention was intended, where possible, to match outreach caller cultural background and main language with that of the patient called, and our study included patients whose main language was not English where this had been achieved. These participants placed high value on receiving an outreach call from a known and trusted member of their community who was able to communicate with them in their own language. There were also data which indicated that a “mismatch” in main language between caller and patient could reduce the effectiveness of the intervention. Our data demonstrate that “matching” went beyond language, with interviews indicating that participants found it helpful if they could identify with the person who called them, such as Sonia, whose caller had spoken about her own and her husband's experiences of NHS Health Checks. Our linked qualitative evaluation with staff delivering the telephone outreach intervention found that “matching” was important—to capitalize on outreach workers’ specialist skills and maximize the potential impact of the intervention. However, such matching could be difficult to achieve due to (a) ethnicity being poorly recorded in medical records,^12^, ^13^ and (b) the support of participating primary care practices being required for outreach callers to be provided with appropriate lists of patients (T. J. Stone, E. Brangan, A. Chappell, V. Harrison, J. Horwood, unpublished data).

An innovative element of the Bristol telephone outreach intervention was the completion of part of the NHS Health Check during the call. While the majority of our participants found this acceptable, the questions were often repeated at the NHS Health Check appointment; thus, one of the intended benefits—saving time during the appointment—may often not have been achieved. Another intended benefit was to instigate a conversation about lifestyle factors relevant to cardiovascular health, and if appropriate provide people with information about local services where they could access support should they wish to make changes—for example, increasing physical activity or giving up smoking. A minority of participants reported that the outreach call had caused them to think, or behave, differently in regard to their physical activity or diet. However, some negative experiences related to this part of the intervention were also reported; thus, the evidence on whether completing part of the NHS Health Check during the call is beneficial is mixed.

A rapid evidence synthesis^3^ found little data on behaviour change or referrals to lifestyle services linked to the NHS Health Check programme, and staff involved in delivery of NHS Health Checks in primary care reported difficulties in influencing people to make long‐standing changes to their lifestyles, and in accessing lifestyle services.^6^, ^14^, ^27^, ^28^, ^29^, ^30^ Research with patients found that most had been given lifestyle advice as part of their NHS Health Check, but many had found this advice too simplistic or generic.^7^, ^21^, ^30^, ^31^, ^32^, ^33^ A telephone outreach approach from a community worker with knowledge of local services could potentially have a role here.

### 4.1 Strengths and limitations

This study was part of a larger evaluation carried out in collaboration with the local authority public health commissioners for NHS Health Checks. The overall project included a quantitative evaluation of the early stages of the telephone outreach intervention,^20^ and a qualitative interview study with staff involved in delivering the intervention (T. J. Stone, E. Brangan, A. Chappell, V. Harrison, J. Horwood, unpublished data). Regular meetings of the project team allowed findings from different aspects of the work to inform ongoing data collection and analysis. The involvement of our local authority collaborators was critical in facilitating access for data collection, and meant findings could be communicated in a timely manner, and discussed more openly, than is possible in many academic studies. This both improved the quality of the research and increased its potential for impact locally.

The views of participants in this interview study are unlikely to be representative of all patients who received a telephone outreach call, as those who took part in an interview had all accepted the invitation for the NHS Health Check. While we wished to include the views of patients who had declined the invitation, this was not achieved.

## 5 CONCLUSIONS

The clearest benefits identified in this research may be achievable with a simpler telephone outreach service—with calls made by primary care practice administrative staff providing information about, an invitation to, and an opportunity to book, an NHS Health Check. Qualitative research indicates that this is an approach some practices are already taking for patients who do not respond to a written invitation.^14^ It would thus be beneficial to pilot and evaluate a simplified telephone outreach intervention.

This approach would however forfeit two important opportunities: engaging groups who might otherwise encounter language or cultural barriers to taking up an invitation to an NHS Health Check and signposting patients to appropriate local lifestyle services.

Future research should thus explore in more detail which patients would benefit from an outreach caller with specialized training, skills or characteristics, and how best to implement “matching” of specialized callers and patients at a local level.

## CONFLICT OF INTEREST

The authors have no conflict of interest to declare.

## Data Availability

Due to confidentiality, and the nature of the consent obtained, the interview transcripts cannot be shared. For further information related to this data set, please contact the first author.
