# Supplementary material for: Patient experiences of telephone outreach to enhance uptake of NHS Health Checks in more deprived communities and minority ethnic groups: A qualitative interview study
Source: Health Expect. 2018 Dec 25;22(3):364–72. doi: 10.1111/hex.12856 (PMC6543263; doi:10.1111/hex.12856)
Supplement: Supplementary file 1 [file HEX-22-364-s001.docx]

|  |  |
| --- | --- |

**NHS Health Checks Study**

**Patient Topic Guide**

**Part A: Introduction, consent and background**

- Thanks, introduce self, re-state purpose of the interview
- Discussion of how interview will be recorded, right to withdrawal, issues of confidentiality, anonymisation and informed consent. (*face-to-face written consent, telephone verbal consent)*
- Verbal consent: *switch* *audio recorder on – Go through each point on consent form verbally.*
- Background information on participant (e.g. age, ethnicity, location)
- Can you start off by telling me how your health is in general?
- Are you currently being treated for any health problems?
- When did you last visit your GP?
- Any family history of cardiovascular disease - heart disease, stroke, kidney problems or diabetes

**Part B: NHS Health check understanding**

- How did you first hear about the NHS health checks? (how did they hear about it, what were their thoughts/feelings about health checks?)
- What was your understanding of the purpose of NHS health checks?
- Had you considered having an NHS Health Check before you had a phone call from the doctor’s Surgery? (previous invite - letter, text invite, outreach event?)

**Part C: Telephone outreach**

- Talk me through what happened when you were phoned about NHS Health Checks?
- What were your initial feelings about being invited in to have a health check over the phone? (first reaction to the call? understanding of purpose? any concerns about answering the call?)
- How did the caller explain health checks? (communication style etc.)
- What made you decide to accept/decline a NHS Health Check? What sort of questions were you asked and how was this? (ask specifically about parts of health check conducted on phone - demographic details/ethnicity, alcohol, physical activity, smoking status, family history of CVD)
- Were you offered any advice or support / signposting to lifestyle services during the call? What? Attitude to this? Take up of services?
- Was there anything you especially liked about the call? Disliked about the call?
- Do you know who was making the call? (were they local / known to you personally / cultural connection / aware that outreach worker compared to call from practice?)
- Reaction to caller? (gender / ethnicity / accent / language, & implications)
- How easy was it for you to talk to the person on the phone about your health? Why?
- Did identity / characteristics of caller have any impact on this? (What aspects and why?)
- Did the conversation you had have any impact on decision (versus if you had just received a phone call to invite you to book an appointment)?
- Would it have made any difference if it was a member of reception/admin staff from the practice who had called you instead?
- Have you made any changes (or thinking about any changes) as a result of the telephone call about the NHS Health Check?
- Is there anything you think would improve the way the phone calls about NHS Health Checks are made?

**Part D: For those that did not attend a health Check**

- Reasons why you declined/ were unable to attend the health check? (e.g. language barrier, lack of time, intrusive, concerns about feeling judged, concerned about the results, unsure about the benefits, lacks confidence or feels uncomfortable about going to local practice)
- What benefits do you think there would be of having one?
- What disadvantages or concerns would you have? (e.g. intrusive, concerns about feeling judged, concerned about the results, unsure about the benefits, lacks confidence in or feels uncomfortable going to local practice, language barriers, time)
- Is there anything that would make it easier for you to attend a health check?
- Is there anything else you would like to add?
- Thank them for their time

**Part E: For those that attended a health check**

- In what ways did you feel that you would benefit from having a health check?
- What was the process for booking the health check once you had made your decision like? Were you able to get a suitable appointment?
- Can you run me through what happened when you had your health check? How did you find it? Was it what you expected from the phone call?
- Who carried it out and what were they like?
- What were your results? (How did you feel about the results? Understanding of results?)
- How did you hear about your results? (same day, letter, phone) – what format? (verbal, print out, both – visual, % risk score)
- What information did you receive about the results? How did you feel about the information you were given about the results? What could have been better? (were the results clearly presented, were they meaningful to the individual? clear about risks?, lifestyle changes?)
- Did you receive any advice about any aspects of your health? [smoking cessation, alcohol, diet, exercise] What information/advice & what did you think/feel about it?
- Have you attended any groups or appointments through information that was given at the health check?
- Have you been able to make any changes following the advice you were given?
- What were the positive aspects of having a health check?
- How did you feel at the end of the appointment?
- Is there anything you would wish to change about the health check experience? What could have been better?
- Was there any follow up after the appointment, or is there any follow up you would like?
- Is there anything else you would like to add?
- Thank them for their time
