# Supplementary material for: Patient experiences of telephone outreach to enhance uptake of NHS Health Checks in more deprived communities and minority ethnic groups: A qualitative interview study
Source: Health Expect. 2018 Dec 25;22(3):364–72. doi: 10.1111/hex.12856 (PMC6543263; doi:10.1111/hex.12856)
Supplement: Supplementary file 2 [file HEX-22-364-s002.docx]

**Coding categories contributing data to higher level themes**

| **Higher level theme** | ***Contributing coding categories** | **Main sub-categories (where applicable)** |
| --- | --- | --- |
| Participants’ backgrounds & contexts (summarised in results introduction ) | Age, Ethnicity, Socioeconomic background, General health, Family health history, Health system contact, Attitude towards health service, When/how first heard of health checks |  |
| Receiving an NHS Health Check invitation by telephone | Acceptability of phone call  Letter invitation versus phone  Why accepted invitation  Appointment booking process | Recognised phone number versus blocked / unknown number  Potential for initial concern as to why “the doctor’s” is calling  Proactive contact / offer of care from health service  Potential for intrusiveness (if call received at ‘bad’ time)  Ability to decide and act (book) immediately  Not having to try and get through to practice by phone to book  Avoiding risk of ‘putting off’ / not getting around to following up  Able to speak to someone / ask questions  Wanting written information about NHS health check  Wanting written / text confirmation or reminder of appointment  Sensible / why not?  Ease – not having to initiate process  Opportunity to address existing health concerns  Straightforward / quick  Appointment availability / suitability |
| Who telephones, and how they communicate | Caller identity  Caller communication strategies  Understanding of health check and why invited | Connection to primary care practice  Languages spoken  Accent  Caller known to patient  Friendly / chatty / polite / professional  Responsiveness to patient  Sharing personal experience  Clarity of information / explanation |
| Completing part of the health check during the outreach call | Doing part of the check on the phone  Referral or advice from phone call  Changes considered based on phone call | Attitude to being asked questions about health on phone  Phrasing of questions and perceived implications (e.g. judgement)  How easy to respond / provide required information  Questions repeated / checked at health check appointment  Verbal signposting to preventative ‘lifestyle’ services / information  Follow up information sent by post  Reflecting on ‘lifestyle’ behaviours  Changes to ‘lifestyle’ behaviours before health check appointment  Reinforcement at health check appointment |

* For simplicity, categories which contributed to more than one theme are listed by their main theme.
